# Feasibility, Effectiveness, and Acceptability of a Telemedicine Neurological Consultation for Drug-Induced Movement Disorders; A Randomized Pilot Study

**DOI:** 10.5334/tohm.1007

**Published:** 2025-05-05

**Authors:** Emily Houston, Amanda G. Kennedy, Terry Rabinowitz, Gail L. Rose, James Boyd

**Affiliations:** 1 Department of Neurology, University of Vermont Medical Center, Burlington, Vermont, USA; 2 Department of Medicine Quality Program, The Robert Larner, M.D. College of Medicine at the University of Vermont, Burlington, Vermont, USA; 3 Departments of Psychiatry and Family Medicine, The Robert Larner, M.D. College of Medicine at the University of Vermont, Burlington, Vermont, USA; 4 Department of Psychiatry, The Robert Larner, M.D. College of Medicine at the University of Vermont, Burlington, Vermont, USA; 5 Department of Neurological Sciences, The Robert Larner, M.D. College of Medicine at the University of Vermont, Burlington, Vermont, USA

**Keywords:** Neurology, telemedicine, drug-induced movement disorder, RE-AIM, mental health

## Abstract

**Background:**

Individuals exposed to neuroleptics are at risk of developing a drug-induced movement disorder (DIMD). Early identification and appropriate management are necessary to minimize the risk of DIMDs worsening and becoming irreversible. Movement disorder neurologists can assist other clinicians in assessing the cause of the DIMD and make care recommendations. The aim of this study was to evaluate if telemedicine can be used to provide a neurological consultation service to patients with DIMDs.

**Methods:**

Patients referred by mental health clinicians (MHCs) in a rural state were randomized to have a neurological consult in-person or through telemedicine. Participants completed two visits with a neurologist and completed surveys about their experience and well-being. MHCs provided feedback on the service through a survey and qualitative interviews.

**Results:**

In the IP group, 79% or participants completed Visit 1 and 71% attended Visit 2, as compared to 86% of participants completing Visit 1 in the TM group and 57% were present for Visit 2. Satisfaction scores were slightly higher for the IP group at both visits. MHCs were satisfied with the consult, reporting that it was helpful and improved care for the patients.

**Discussion:**

Providing a consult service through telemedicine is feasible, effective, and acceptable, and can be improved further following feedback provided by the MHCs. Overall, participants and MHCs were pleased to have the opportunity to meet and collaborate with a neurologist.

**Highlights:**

The results from our study address gaps in knowledge related to providing specialist care to people with drug-induced movement disorders in a rural setting. Telemedicine consultation is feasible, with low rates of technological issues, and participants with drug-induced movement disorders were satisfied with telemedicine as a mode of care delivery.

## Introduction

Drug-induced movement disorders (DIMDs) are uncontrollable, spontaneous, and often disruptive hypokinetic or hyperkinetic movements, most commonly caused by the use of dopamine receptor blocking agents (DRBAs), primarily antipsychotics, but have also been linked to serotonin reuptake and serotonin norepinephrine reuptake inhibitors, calcium channel blockers, lithium, and illicit substance use [1,2]. There is a long-standing history of antipsychotic therapies causing extrapyramidal symptoms (EPS), beginning with reserpine in the late 1940s [3]. Initially, abnormal movements were thought to be associated with the antipsychotic effects of the first-generation antipsychotics (FGAs), and it was challenging to differentiate EPS such as akathisia from possible psychopathology, like agitation. In 1987, it was recommended that clinicians first rule out the possibility of EPS in patients taking a neuroleptic agent before attributing the symptoms to behavioral changes [4]. The frequently observed and burdensome EPS related to FGAs prompted efforts to develop better tolerated antipsychotics, leading to second and third generation antipsychotics (collectively, SGAs), which are partial dopamine D2 agonists and carry other favorable pharmacodynamic features [5].

Expanded on- and off-label use of SGAs and other DRBAs has contributed to a high prevalence of DIMDs [1,2,6]. A meta-analysis of 41 randomized controlled trials (RCTs) conducted from 2000 to 2015, found the global mean prevalence of tardive dyskinesia (TD), a common DIMD characterized by hyperkinetic movements, to be 25.3% among the 11,493 patients [7]. The rates for TD were lower for SGA (20.7%) than FGA (30.0%) treatment, but not negligible [7]. In the Netherlands, a longitudinal study published in 2017 investigated the prevalence, incidence, and persistence of DIMDs in 1120 younger patients (mean age 27.1 years) with nonaffective psychosis [8]. The mean illness duration for these patients was 4.3 years, and many were primarily treated with SGAs. Of the 828 patients who developed a DIMD, the most prevalent was parkinsonism (32%), followed by akathisia (9%) and TD (4%). During the three-year study period, and while participants received antipsychotic treatment, it was reported that the persistence of these DIMDs was relatively high, with parkinsonism persistence rates at 53%, akathisia at 17%, and TD at 20%. The authors concluded that even in a younger population with a recent onset of psychosis and treatment with SGAs, it is important to monitor for DIMDs [8].

Clinicians have agreed that prevention of DIMDs is imperative, as they can become persistent, disruptive, and irreversible [9,10,11]. In a study comparing patients with schizophrenia, bipolar disorder, and major depressive disorder, it was found that patients with TD reported significantly worse health-related quality of life and social withdrawal, and the severity of the movement disorder was associated with greater decline of these outcomes [12]. To reduce the deficit in physical and mental functioning, clinicians must accurately diagnose the DIMD, determine which medication has contributed to the symptoms, and select appropriate treatment options [13]. Determining the diagnosis has been challenging for clinicians, due to overlapping presentations of certain DIMDs, limited knowledge about the spectrum of symptoms for each disorder, not enough general training focusing on DIMDs, and delayed symptom reporting by patients who may be ashamed or lacking insight [9,11,14]. Psychiatrists and other mental health clinicians (MHCs) managing DIMDs have been tasked with making a number of pharmacological decisions that may require multiple steps for their patients, such as reducing or changing their antipsychotic treatment, or adding on adjunctive therapy [13]. Collaborating with a movement disorder specialist for an in-depth evaluation is advised, as some of the differential diagnoses of TD and parkinsonism are rare and not routinely seen in psychiatric practice [9,13].

While including a movement disorder specialist is recommended for diagnosing and providing treatment recommendations, there are currently issues impacting access, such as an increase in patient referrals, a nation-wide shortage of neurologists, and more specialists based in urban settings [15,16]. Telehealth has been widely studied in movement disorders, particularly Parkinson’s disease (PD), and saw a massive expansion of use in psychiatry during and after the COVID-19 pandemic, which could potentially remediate some barriers to specialty care [17,18]. For instance, among patients experiencing hyperkinetic movement disorders, many of the features of tremor, tics, dystonia, dyskinesia, and myoclonus have had the potential to be evaluated through telehealth [19]. However, there may be limitations with video quality, symptoms not being present at the time of the event, standardized assessments needing modification to accommodate a remote exam, and other site-specific challenges that require further investigation [19]. The robust research and clinical experience with telemedicine in PD has led to a growing interest in the feasibility, effectiveness, and acceptability of remotely evaluating other movement disorders [19,20]. This randomized pilot study aims to contribute to the literature by comparing in-person (IP) to telemedicine (TM) neurological consultation visits for patients suspected to have a DIDM, and to investigate the feasibility, effectiveness, and acceptability of a statewide neurological consultation service especially in the context of multidisciplinary care across a rural state.

## Methods

### Trial Design

Participants were referred to the study by their MHC, who agreed to provide feedback throughout the study. Participants were randomized 1:1 to the IP or TM group. Implementation of the service was evaluated using the Reach, Effectiveness, Adoption, Implementation, and Maintenance (RE-AIM) framework and mixed methods. In this article, we have presented on the RE-AIM components and outcomes related to reach, effectiveness, and acceptability. This study was performed in accordance with the Declaration of Helsinki and approved by the University of Vermont Institutional Review Board (STUDY00001579). All participants were consented using an electronic informed consent form.

This study was registered at clinicaltrials.gov, NCT06060444.

### Participants

Participants were eligible if they had a suspected DIMD or had received a DIMD diagnosis, but diagnostic confirmation and treatment recommendations were requested, based on documentation in a referral note. They needed to be referred by a MHC that was agreeable to participating in the study by contributing data related to themselves, their practice, and their experience with the consultation service. Participants and MHCs were located throughout the state of Vermont, and at the time of consent, participants needed to be able to travel to the University of Vermont Medical Center for in-person visits and have the ability to connect to the videoconferencing application, Zoom (Zoom Video Communications, Inc., San Jose, CA). Eligible participants were 18 years or older, willing to complete surveys after each visit, and not currently hospitalized.

### Interventions and Procedures

The Neurology study team (NST) compiled a list of Vermont’s mental health agencies, as well as other community and private mental health practices, to send information about the study. As part of recruitment and outreach efforts, the NST sent introductory emails or spoke on the phone with medical directors, support staff, and the MHCs. The NST offered to present the study at staff meetings, virtual meetups with groups or individual MHCs, and provided recruitment materials for their patients or to display in clinic.

Participants that were referred to the study and interested in participating were sent a link via email to the electronic consent form, along with a unique code. Once consent was obtained, the NST contacted the MHC for clinical notes, and their impression of the participant’s movement disorder severity, using the Clinical Global Impression-Severity (CGI-S) scale. The groups were stratified based on the GCI-S score, with 1–4 as lower severity and 5–7 as higher severity, before participants were randomized to the IP or TM arm. Participants were then scheduled to have their initial consult visit (Visit 1) with a movement disorder specialist at our Neurology clinic, or through Zoom. The TM participants were sent a link to the Health Insurance Portability and Accountability Act- compliant platform, Zoom (Zoom Video Communications, Inc., San Jose, CA), either through email or their electronic health record patient portal. They were able to receive technical support from case workers, other clinicians, hospital information technology, the NST, or family and friends, if needed. The neurologist performed a set of assessments specific to the reported symptoms and in-line with standard examinations (e.g. AIMS, MDS-UPDRS), and modified for the remote group. They documented their findings and shared them with the MHC. If more information was needed, they would arrange a phone call or discuss the case through secure written communication. Following Visit 1, participants were emailed a REDCap link to complete surveys which included a demographics questionnaire, the Clinical Satisfaction Questionnaire (CSQ-8), and the Patient Reported Outcomes Measurement Information System (PROMIS^®^29+2 Profile V2.1) [21,22,23].

Participants returned a few months later for Visit 2, either in-person or remotely. The same neurologist repeated examinations of their abnormal movements and evaluated if there were any changes to physical or mental health. They determined if their treatment recommendations were applied, to what extent, and if they’d advise on any additional care changes. The neurologist documented their findings to again share with the MHC. The participants were sent a second REDCap link to electronically complete the CSQ-8 and PROMIS^®^29+2 Profile V2.1 following their visit.

The NST conducted two rounds of qualitative interviews with the MHCs who had referred enrolled participants. The interview asked about satisfaction with the consultation service, and other components of RE-AIM (adoption, implementation, and maintenance). The interviews were completed over Zoom with audio recording, then converted into a written transcript using Microsoft Word. At the end of the study, the MHCs were sent an electronic six-item survey that included a question about overall satisfaction.

### Outcomes

Feasibility was measured by comparing the proportion of participants who successfully completed at least Visit 1, and the proportion of participants who completed Visit 2. We compared the visit completion rates between the two groups, along with the proportion of surveys that were completed after Visits 1 and 2. Any technical issues that affected the occurrence or quality of a visit were summarized for the TM group, while any barriers to arriving for an in-person visit were also reported.

Effectiveness, as defined by the impact on patient health status, was quantified by the change in PROMIS^®^29+2 Profile V2.1 scores from Visit 1 to Visit 2. We also measured the rate of concordance between the DIMD symptoms reported by the MHC and confirmation or recategorization by the movement disorder specialist. We evaluated the impacts to the care plan in these categories: medication changes, non-pharmacological treatments, and lifestyle changes. Effectiveness was also measured from the MHC’s perception of improvement, using the MHC survey, question #3 “Did the consultation improve care for the patient?”

Acceptability for both the participants and the MHCs was quantified using satisfaction measures. We compared the participant-completed CSQ-8 means between the IP and TM groups. The MHC satisfaction survey allowed us to determine the proportion of MHCs that were satisfied, based on the number of positive responses to the six questions.

### Sample Size

For this pilot study, the sample size required to determine a statistical significance between the two groups for the CSQ-8, using previously reported differences in means and standard deviations was not feasible for this project [24]. We decided an enrollment goal of 28 participants, assuming a 21% dropout rate, would result in 12 participants in each arm. This is a small sample size designed to capture preliminary, pilot data, and it does not ensure adequate power to detect a true effect.

### Randomization

The study’s Principal Investigator (EH) generated a random allocation sequence using Excel, with block sizes of 4, then uploaded it into REDCap. Once the consent form was signed, participant data was entered into REDCap, including the CGI-S for stratification. This produced the unblinded group assignment, which was shared with the participants, the movement disorder specialist, and the MHC.

### Statistical Methods/Analysis

Feasibility outcomes were summarized using descriptive statistics. The percentages of completed visits and surveys for both groups were calculated and compared. Within the TM group, the percentage of visits with technology challenges was calculated. The reported reasons for missing an in-person visit were collected and presented as nominal categories with proportions. The characteristics of the participants in the two study groups were compared using a Fisher’s exact test (for dichotomous variables) or a t-test (continuous variables).

To analyze effectiveness as impact on patient health outcomes, each of the PROMIS^®^29+2 Profile V2.1 eight domains (physical function, anxiety, depression, fatigue, sleep disturbance, satisfaction with participation in social roles, pain interference, and cognitive function) had the raw score calculated per participant and converted into *T* scores. The *T* scores were used to calculate the mean for each domain within the IP and TM groups. The non-normally distributed PROMIS^®^29+2 Profile V2.1 data were analyzed using the Mann-Whitney U test for both Visit 1 and 2. The Wilcoxon signed rank test was used to examine the difference in paired mean *T* scores of each domain for the repeated PROMIS^®^29+2 V2.1 measure within both groups. Due to multiple comparisons, a Bonferroni correction was used to adjust the significance level from 0.05 to 0.006. For symptom concordance, the terms or diagnosis in the MHC notes from prior to the study were compared to the main symptoms or diagnosis reported by the neurologist to determine a dichotomous yes/no outcome. The proportion of diagnoses that were in agreement were compared between groups. The changes to the care plans were summarized by creating nominal categories for each change and presenting the rates study-wide. The proportion of MHCs who responded positively on question 2 of the MHC survey, asking “Did the consultation improve care for the patient?” was calculated.

The satisfaction measures were a proxy for acceptability. The mean scores were calculated for the CSQ-8 and compared between the IP and TM groups using a Mann-Whitney U test. We used two-tailed significance tests and set alpha at 0.05 when running the statistical analyses. We determined the percentage of MHCs that were satisfied with the consultation program, based on the number of positive responses to the six questions of MHC survey. The qualitative interview data were coded in grounded theory by a member of the NST (EH) who individually open coded the responses, then compared the data and identified concepts and categories. Axial coding allowed for organization of the categories to identify connections and similar themes, which were used to develop a theory about the acceptability of a neurological consult service from the MHCs’ perspectives.

## Results

### Feasibility

**Figure 1:**
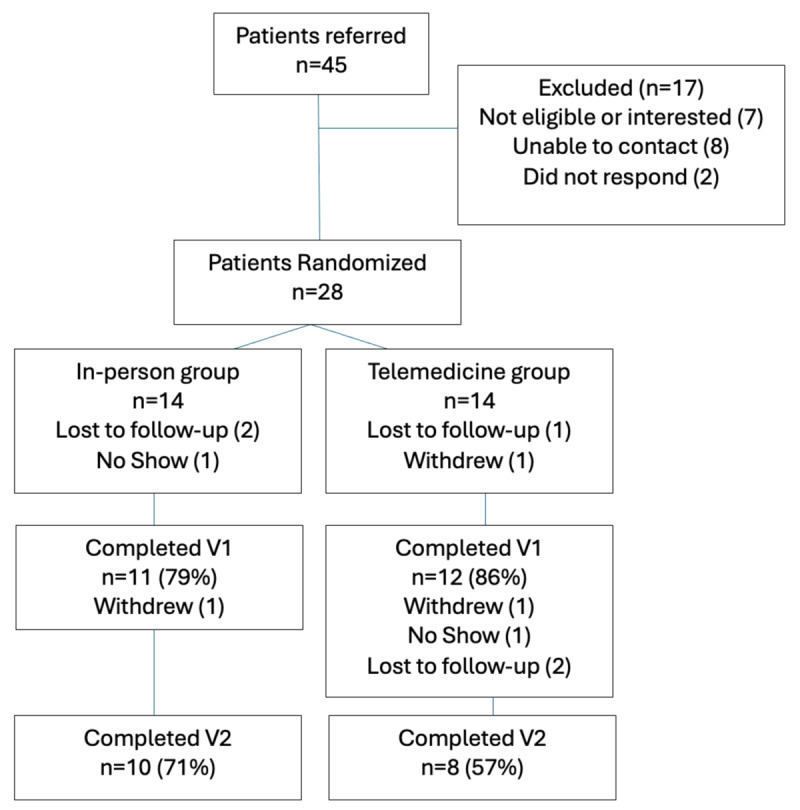
Flow diagram of Study Participants.

A total of 45 patients were referred to our study from twenty MHCs at eleven practices throughout the state of Vermont (Figure 1). Half (10/20) of the MHCs were psychiatrists, and half were nurse practitioners. Twenty-eight patients were randomized to one of the two arms, but after five people were either lost to follow-up, withdrew, or no-showed for Visit 1, 23 (51% of referred) participants successfully completed Visit 1. In the IP group, three out of fourteen participants withdrew before Visit 1 (79% completed), while twelve out of fourteen (86%) of participants in the TM group completed V1. Visit 1 surveys were completed by 11 IP participants (79%) and 10 TM participants (71%). Between Visit 1 and 2, one person in the IP group withdrew, leaving 10 participants (71%) to complete the visit and surveys; while the TM group had two people drop-out, resulting in only 8 participants (57%) completing Visit 2 and surveys. Only one participant in the TM group withdrew because they did not have sufficient video quality during the synchronous call. No one else in the TM reported difficulty with joining the remote appointment or with technical challenges.

Participants were mostly female (IP = 73%, TM = 80%), white (IP = 82%, TM = 100%), and at least half had completed a bachelor’s degree or higher (IP = 64%, TM = 50%). The two groups only varied significantly on marital status, as shown in Table 1. A large proportion of both groups had a history of schizophrenia (IP = 45%, TM = 40%), and a range of other psychiatric disorders, including bipolar affective disorder I or II (IP = 37%, TM = 20%) and major depressive disorder (IP = 9%, TM = 30%). Other psychiatric disorders that participants experienced were anxiety, attention-deficit/hyperactivity disorder (ADHD), substance use disorders, and post-traumatic stress disorder (PTSD). The most common movement disorder diagnosis was TD (IP = 45%, IM = 80%). Table 1 provides the frequencies of the movement disorder diagnoses, with multiple participants presenting with mixed movement disorders.

**Table 1:** Participant Characteristics at Visit 1.


	IN-PERSON n = 11	TELEMEDICINE n = 10	p-VALUE

**Age**	52.4 (15.1)	58.7 (8.7)	0.26

**Female [n(%)]**	8 (73)	8 (80)	0.34

**Race [n(%)]**			0.13

Caucasian	9 (82)	10 (100)	

Other	2 (18)	0 (0)	

**Bachelor’s degree or higher [n(%)]**	7 (64)	5 (50)	0.21

**Marital status [n(%)]**			0.05

Never married	3 (27)	6 (60)	

Currently married	5 (45)	0 (0)	

Divorced	2 (18)	4 (40)	

Widowed	1 (9)	0 (0)	

**Employment Status [n(%)]**			0.46

Employed Full or Part time	4 (37)	3 (30)	

Retired	5 (45)	4 (40)	

Unemployed	2 (18)	3 (30)	

**Insurance Type [n(%)]**			

Private insurance	4 (37)	3 (30)	0.34

Medicaid or Medicare	6 (54)	7 (70)	

Other	1 (9)	0 (0)	

**Distance from Neurology Clinic [miles (mean (SD))]**	17.2 (15.9)	22.5 (16.9)	0.55

**Psychiatric Disorders [n(%)]**			

Schizophrenia	5 (45)	4 (40)	

BPAD I or II	4 (37)	2 (20)	

MDD	1 (9)	3 (30)	

Other	6 (54)	4 (40)	

**Movement disorder diagnosis [n(%)]**			

Tardive dyskinesia	8 (80)	5 (45)	

Drug-induced parkinsonism	2 (20)	3 (27)	

Drug-induced tremor	3 (30)	2 (18)	

Drug-induced akathisia	1 (10)	1 (9)	

Primary parkinsonism	0 (0)	1 (9)	

Other	3 (30)	3 (27)	

Unable to assess	0 (0)	1 (9)	


Abbreviations: BPAD I or II = Bipolar I disorder, Bipolar II disorder; MDD = major depressive disorder.

### Effectiveness

At Visit 1, the IP and TM groups were very similar on levels of anxiety, depression, satisfaction with participation in social roles, and cognitive function, and were not significantly different on any of the eight domains (Table 2). After the second visit, there was more variation on the *T* scores for physical function and sleep disturbance, but there was no significant difference between any of the PROMIS^®^ outcomes. There was not a significant difference in means from scores at Visit 1 to 2 for all domains. Interestingly, satisfaction with participation in social roles increased for the IP group and decreased for the TM group (IP = 3.78, TM = –3.99, p = 0.02). Three other outcomes had changes in means that went in different directions between the two groups: physical function (IP = 0.89, TM = –1.27), depression (IP = –1.46, TM = 0.96), and cognitive function (IP = 1.60, TM = –1.89).

**Table 2:** Change in Efficacy and Acceptability Outcomes Among Patient Participants.


	V1 (n = 21)			V2 (n = 18)			DIFFERENCE IN MEANS (PAIRED)**		DIFFERENCE IN MEAN CHANGE	
	
	IP (n = 11)	TM (n = 10)	p-VALUE*	IP (n = 10)	TM (n = 8)	p-VALUE*	IP (n = 10)	TM (n = 8)	p-VALUE*	

**CSQ-8**	29.91 (4.10)	27.50 (5.89)	0.25	30.50 (2.27)	24.25 (5.90)	0.01	0.8 (–1.27, 2.87)	–3.12 (–6.57, 0.32)	0.02	3.92

**PROMIS^®^29+2 Profile V2.1**										

Physical function	48.52 (9.83)	40.40 (9.44)	0.10	50.38 (9.24)	41.52 (7.72)	0.06	0.89 (–3.56, 5.34)	–1.27 (–6.27, 3.72)	0.34	2.16

Anxiety	56.95 (10.71)	56.78 (10.14)	0.92	54.79 (8.90)	55.45 (8.32)	0.93	–0.73 (–8.00, 6.54)	–2.09 (–5.37, 1.19)	0.42	0.54

Depression	53.95 (10.60)	55.62 (7.34)	0.94	51.46 (8.44)	56.16 (8.38)	0.21	–1.46 (–7.62, 4.70)	0.96 (–2.21, 4.14)	0.26	2.42

Fatigue	54.11 (6.60)	53.48 (7.18)	0.60	49.57 (7.19)	52.14 (9.49)	0.72	–3.48 (–9.97, 3.00)	–3.21 (–9.48, 3.06)	0.96	0.27

Sleep disturbance	52.29 (10.88)	56.26 (9.56)	0.42	46.73 (6.35)	54.59 (8.65)	0.07	–5.47 (–13.29, 2.35)	–4.42 (–10.53, 1.68)	0.79	1.05

Participate social roles	46.76 (11.58)	46.52 (9.57)	0.83	52.05 (10.12)	45.20 (7.33)	0.15	3.78 (–1.79, 9.35)	–3.99 (–7.52, –0.45)	0.02	7.77

Pain interference	52.15 (11.32)	58.43 (8.94)	0.19	51.49 (11.21)	57.57 (8.51)	0.22	0.67 (–4.51, 5.85)	2.01 (–3.11, 7.13)	0.86	1.34

Cognitive function	48.24 (8.48)	49.23 (7.67)	0.80	50.56 (6.26)	46.37 (5.91)	0.30	1.60 (–3.76, 6.96)	–1.89 (–9.51, 5.74)	0.44	3.49


Abbreviations: CSQ-8 = Client Service Questionnaire 8-item; PROMIS^®^ = Patient Reported Outcomes Measurement Information System. Visits 1 and 2 values are mean (SD); Difference in means (paired) are means (95% CI). *Independent samples test. **Dependent samples test.

In both groups, there was high concordance between the diagnosis or description of the motor symptom phenotype given by the MHC and the neurologist (IP = 73%, TM = 92%). The neurologists were certain of their diagnosis in all but the one case where they experienced technical difficulties and could not adequately assess the participant. The most common recommendations were to reduce or stop taking a medication (IP = 82%, TM = 42%), and to add a new medication (IP = 54%, TM = 67%). The frequencies of pharmacological and non-pharmacological recommendations are provided in Table 3. When asked “Did the consultation improve care for the patient?” 63% of MHCs responded either “yes, a lot” or “yes, a little” and the remainder (37%) were neutral; no negative responses were given.

**Table 3:** Diagnostic Concordance and Care Recommendations.


	IP (n = 11)	TM (n = 12)

**Diagnosis Concordance, Yes [n(%)]**	8 (73)	11 (92)

**Impact on Care**		

**Medications [n(%)]**		

Reduce/stop	9 (82)	5 (42)

Add new med	6 (54)	8 (67)

No change	1 (9)	3 (25)

**Diagnostic/referrals [n(%)]**		

Imaging	2 (18)	2 (17)

Labs	1 (9)	1 (8)

EMG/NCS	1 (9)	1 (8)

Sleep eval/SLP swallowing eval/OT	2 (18)	2 (17)

Genetic testing	1 (9)	0 (0)


Abbreviations: EMG = electromyography; NCS = nerve conduction study; OT = occupational therapy; SLP = speech-language pathology.

### Acceptability

**Figure 2:**
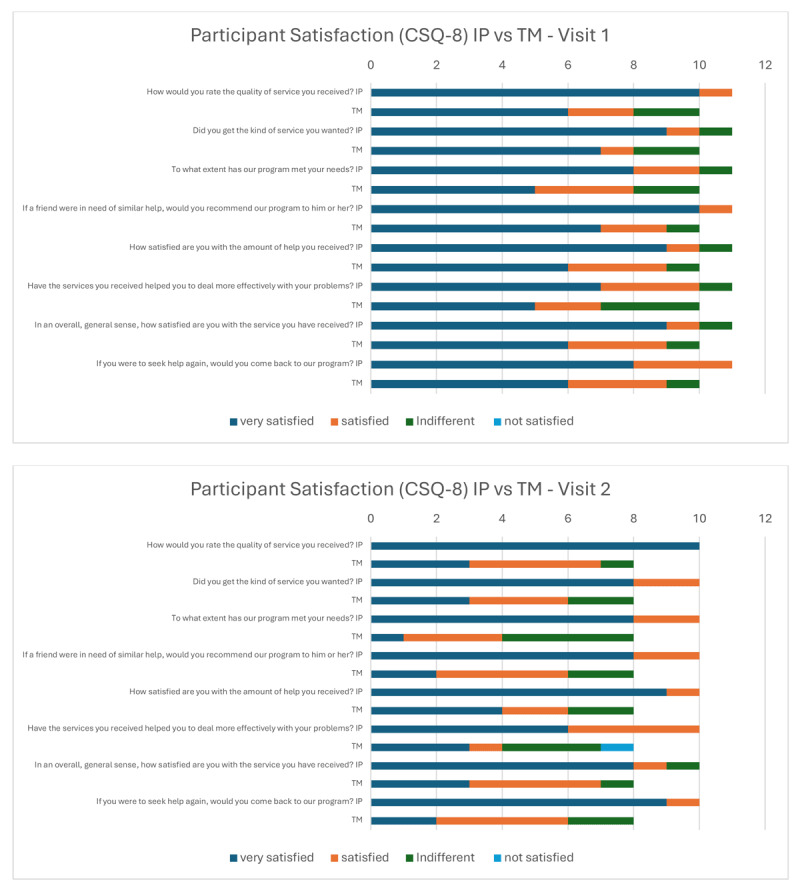
Participant CSQ-8 Responses Per Group at Visit 1 and 2.

Following Visit 1, the mean CSQ-8 scores were fairly high among the two groups (IP = 29.91, TM = 27.50) (Table 2). The proportion of responses is shown in Figure 2, with most participants reporting high satisfaction or agreement, one person in the IP group was indifferent, and one to three people were neutral on a few questions in the TM group.

**Figure 3:**
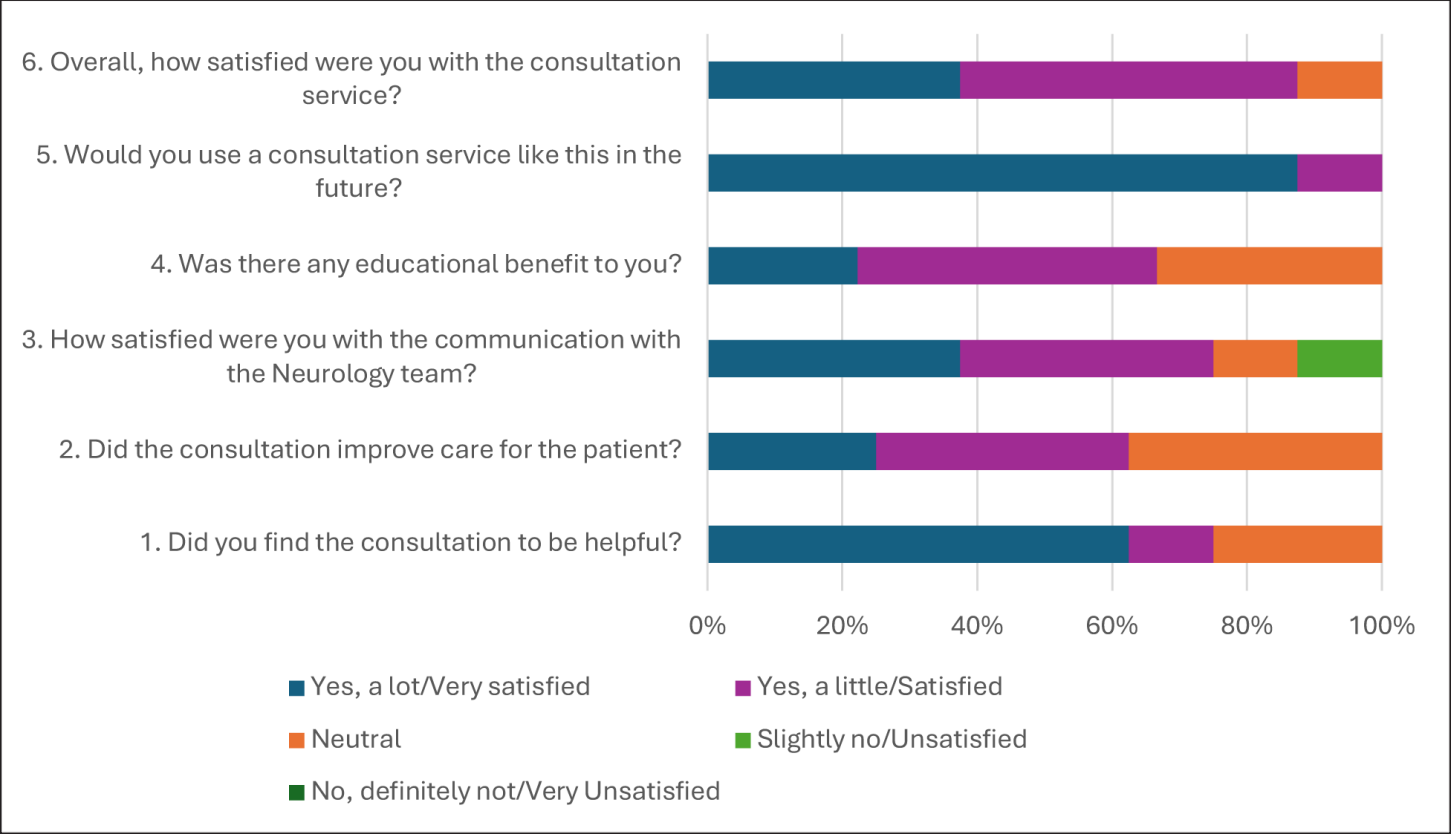
MHC Survey Responses.

The IP group had an increase in CSQ-8 scores from Visit 1 to 2 (Mean diff = 0.8), but the TM group had a –3.12 decline in score (Figure 2). In both groups, the confidence interval crosses zero, indicating that there is not enough evidence to support an effect. Comments at the end of the survey were all very positive in the IP group, stating how excellent and helpful the service was for their care, while comments in the TM group were mixed between statements of benefit and support, to frustration of their health problems still existing, or having to wait for the neurologist to join the Zoom call. The majority of MHCs (n = 8) responded favorably to each question of the MHC survey (Figure 3). All MHCs agreed that they would use a consultation service like this in the future, and over 60% agreed strongly that the consultation was helpful. Two themes emerged from the qualitative interviews, regarding acceptance within the MHCs’ practice and among patients. The first was resources, as related to their staffing support, their workload, and the ability to easily share information with the neurology team. A second was patient factors, which was built off categories such as complex care needs, mistrust of healthcare professionals, and unwillingness to change medications.

## Discussion

Remote consultations in neurology and psychiatry have expanded since the COVID-19 pandemic and TM is widely used in rural areas [25]. This pilot study investigated the potential for TM consultation visits to be feasible, effective, and accepted, when comparing it to IP visits. To our knowledge, this is the first prospective randomized study to compare IP and TM visits among patients with DIMDs.

Previous studies examining TM use for movement disorders, primarily PD, have reported higher rates of attended visits; but earlier studies utilizing telepsychiatry and comparing this method to IP consultation had similar rates of visit adherence to our project, above 70% [20,26,27]. Our Visit 1 attendance rates were good for this population in both groups, with 79% of participants in the IP group and 86% in the TM group. The IP group saw better retention at follow-up than the TM group (71% versus 57%), as the latter had four participants drop-out before Visit 2. Two of the TM participants that dropped out were not engaged, as indicated by noncompliance with completing the Visit 1 surveys, and attempts to communicate with them following their visit were unsuccessful. Another TM participant experienced technical difficulties at their first visit, with poor camera quality and resolution. They opted not to retry a remote visit, and preferred meeting with a neurologist closer to where they lived. This was the only case of technological challenges affecting the neurologist’s ability to complete an assessment. In general, the two neurologists felt confident performing an examination remotely, even in the absence of testing tone, strength, and postural stability. There is concern that patients with disabilities, low health literacy, or living in low socioeconomic areas may have reduced access to equipment or other resources necessary for TM visits, but we found that the majority of participants in this study had access to the necessary technical tools- a working camera and microphone, email or access to an EMR account, and internet [28]. Our findings suggest that TM is a feasible method of performing neurological consults for people with DIMD, and the likelihood of patients attending the visits is influenced by their willingness to engage, rather than technological or clinician concerns.

We used the PROMIS^®^29 instrument to measure and compare physical and psychological health statuses of the study participants; the values give a sense of the participant’s health compared to the US general population [22]. For each PROMIS^®^29 domain, 50 is the average score for the general population and 10 is the standard deviation [29]. A higher *T-*score represents more of what is being measured, for instance, a higher score for depression indicates more depression, while a higher score for physical function means the respondents can function more. At Visit 1, the participants were similar in all domains, except the TM group had more pain interference and scored lower on physical function. We did not see any robust changes in mean values for the health outcomes, other than sleep disturbance, which was the only domain to change by five points, or half a standard deviation [30]. The IP and TM groups both showed an improvement in having less sleep disturbance, though this effect was not consistent for all participants based on the confidence interval estimated for our sample. Both groups had a slight decrease in anxiety and fatigue, but demonstrated opposing changes from Visit 1 to Visit 2 across other domains. Interestingly, the ability to participate in social roles improved nearly four points for the IP group but worsened by four points in the TM group. The *T-*score was below the US general population average at Visit 1 for both groups, but the IP group saw an improvement, which could have been related to medication adjustments and a change in their unwanted movements. Overall, there was no clinically meaningful change in health-related quality of life for participants seen remotely, in either direction. We wouldn’t expect health outcomes to change dramatically in this short period, based on the care recommendations that can take time to implement and monitor for the effects. In just the TM cases, the neurologist recommended a reduction or discontinuation of a current medication to 42% of the participants. The initiation of a new medication, such as a vesicular monoamine transporter type 2 (VMAT2) inhibitor, was recommended for 67% of the TM participants. As many of the adjusted medications were for psychotic disorders, it was important to ensure that these changes did not cause detrimental effects. It appears that we were able to effectively make care recommendations through TM without negatively affecting health-reported wellbeing. Additionally, a large proportion of the MHCs at the end of the study reported that the consultation improved care for their patient(s), while close to a third were neutral.

We saw high levels of satisfaction in both groups following Visit 1, with 90–100% of participants in the IP group reporting that they were “satisfied” or “highly satisfied” on each of the eight CSQ-8 questions, and 70–90% of TM participants also gave satisfied responses on the CSQ-8. Interestingly, the three TM participants that gave the lowest scores on the CSQ-8 had the lowest T-scores on the physical function and ability to participate in social roles and activities domains of the PROMIS^®^29. While the IP participants remained highly satisfied in Visit 2, there was a three-point decline in mean CSQ-8 score for the TM group. This was largely due to mixed responses in a small sample, where half of the participants reported satisfaction with the information they were given and the virtual format, but the other half was indifferent. One comment from a TM participant was that they had a “generally ok experience, but problems still existing.” This illustrated that the delivery of care through TM is acceptable to patients, but our study participants had not gotten the movement disorder symptom resolution they had hoped for by the second visit. The MHCs were accepting of a consultation service and noted that the TM option gave their patients flexibility and made it easier for those without transportation. To support a consultation service in the future, MHCs suggested that we need to identify easy and secure ways to share information and have a designated contact person for them and their patients. It’s important to consider the complex care needs of patients with DIMDs and how those needs may impact their ability to attend multiple visits, as well as their reluctance to adjust or change medications that have kept their psychiatric disorders stable. Based on the responses from the MHCs and participants, they are accepting of TM visits but would like the option to choose between remote or in-clinic visits, based on their needs and comfort levels.

This study was limited by recruitment challenges, such as high staff turnover within the mental health practices, which impacted the availability of the MHCs to engage and required us to re-educate new clinicians and staff on the project. Also, MHCs reported that their patients were hesitant to join a research study, due to concern over medication changes, mistrust of unknown healthcare providers, or assuming that they would be asked to take an investigational drug. Another recruitment limitation was the inability for participants to choose the method of care delivery, as many were without transportation, or only wanted an IP visit. Our study was limited by its small sample size, that it was conducted at one neurology clinic in a non-diverse region, and that the MHC agencies and participants were located relatively nearby. A future study could address this by having multiple neurology sites, each interacting with mental health practices in their area, casting a wider net for recruitment. Low response rates on the surveys by both the participants and MHCs limited the feedback, as did our decision not to survey MHCs or participants that did not participate. Including them might have helped to better understand why they chose not to join, or what potential barriers they faced. Our effectiveness results were limited by the short follow-up period, and extending this timeline to add assessments further out would give more opportunity to determine if the care recommendations influenced health outcomes. A limitation of the design was lack of comparison between an IP and TM evaluation for the same participant, and inter-rater concordance was not tested. Lastly, we did not include a clinical measure, like a standardized motor exam, which could be used in future studies to measure changes in motor symptoms.

## Conclusion

There is a high demand for specialist care in neurology and TM could minimize barriers for patients to be evaluated by a neurologist. A consult service through TM is feasible, based on the low rates of technological issues and the ability of the neurologists to assess the participants remotely. The neurologists were effectively able to make recommendations to participants through TM, without any negative impacts on their health statuses. We cannot endorse that TM is as effective at providing care and improving outcomes in this population as IP visits, due to the small sample size. Telemedicine as a mode of care delivery is acceptable, based on satisfaction scores and feedback from participants, but further research will need to investigate if this patient population prefers to receive information and care in a clinic setting, remotely, or as a combination of IP and TM visits.
